# HIV burden in men who have sex with men: a prospective cohort study 2007–2012

**DOI:** 10.1038/srep11205

**Published:** 2015-07-02

**Authors:** Zhongwei Jia, Xiaojie Huang, Hao Wu, Tong Zhang, Ning Li, Peipei Ding, Yixuan Sun, Zhiying Liu, Feili Wei, Hongwei Zhang, Yanmei Jiao, Yunxia Ji, Yonghong Zhang, Caiping Guo, Wei Li, Danlei Mou, Wei Xia, Zhen Li, Dexi Chen, Huiping Yan, Xinyue Chen, Jinkou Zhao, Kathrine Meyers, Ted Cohen, Kenneth Mayer, Joshua A Salomon, Zuhong Lu, Christopher Dye

**Affiliations:** 1 National Institute of Drug Dependence, Health Science Center, Peking University Beijing 100191, China; 2 Peking University Clinical Research Institute, Health Science Center, Peking University Beijing 100191, China; 3 Takemi Program in International Health, Global Health and Population, Harvard T.H. Chan School of Public Health, Boston, MA, 02115, USA; 4 Center for Infectious Diseases, Beijing You’an Hospital, Capital Medical University, Beijing 100069, China; 5 School of Geography, Beijing Normal University, Beijing 100875, China; 6 The Global Fund to fight AIDS, Tuberculosis and Malaria, 1214 Geneva, Switzerland; 7 Aaron Diamond AIDS Research Center, New York, 10016, USA; 8 Epidemiology, Harvard T.H. Chan School of Public Health, Boston, MA, 02115, USA; 9 The Fenway Institute, Fenway Health, Boston, MA 02118, USA; 10 Global Health and Population, Harvard T.H. Chan School of Public Health, Boston, MA, 02115, USA; 11 Department of Biomedical Engineering, College of Engineering, Peking University, Beijing 100871, China; 12 HIV/AIDS, Tuberculosis, Malaria & Neglected Tropical Diseases, World Health Organization, 1211 Geneva 27, Switzerland

## Abstract

We conducted a prospective cohort study among HIV-negative MSM aged 18 years or older between 2007 and 2012 in Beijing, China to measure the rates of incident HIV and identify risk factors for infection. Among 5,800 participants evaluated at enrollment, we identified 486 prevalent cases of HIV (8.4%). Among the 3,625 enrollees who were HIV-negative at enrollment and completed at least one follow-up interview, we identified 440 incident cases of HIV in the follow up period: this constituted an HIV incidence rate of 7.1 per 100 person-years (95% CI: 6.4–7.7). Early treatment of syphilis may have significantly reduced risk of HIV infection (RR: 1.45, 95% CI: 1.11–1.93), while MSM presenting perfect compliance in the cohort did not show reduction in HIV infection. Our study suggested that HIV incidence has been remained high in this sample of Chinese MSM during the intensive preventive intervention, suggesting that we need to find new strategies to prevent HIV infection in this population.

Although the global HIV epidemic is declining, men who have sex with men (MSM) remain at high risk of infection^1^,^2^,^3^,^4^,^5^. Despite this risk, the epidemic among MSM is relatively poorly understood in many settings because these men are so hidden that they are generally underreported in population-based surveys^6^,^7^,^8^. Incomplete knowledge about the risks experienced by this vulnerable population limits the identification of effective interventions.

Consistent with this global trend, data from China show that the proportion of reported HIV cases infected through sexual contact has increased from 33% in 2006 to 76% by 2011, of which 14% cases were attributable to MSM. This number was still lower, however, than the 17% figure estimated in 2011^9^,^10^. A meta-analysis of HIV incidence in MSM using nationwide data estimated an HIV incidence of 3.5 per 100 person-years (95% confidence interval (CI): 1.7–5.3) within prospective cohort studies of seronegative individuals and 6.7 per 100 person-years (95% CI: 4.8–8.6) for cross-sectional studies^11^. A 1-year cohort study in 2010 reported that HIV incidence was 8.1 per 100 person-years (95% CI: 6.92–9.26) in Beijing^12^. Recently, another study using nationwide surveillance data and a systematic review estimated that HIV incidence in MSM might be as low as 0.98 per 100 person-years (0.70–1.25) in China in 2010^13^.

Here we describe a 5-year prospective cohort study among MSM in 16 districts of Beijing, China, in which we assess the baseline HIV burden, HIV incidence and also evaluate the impact of condom promotion and education on condom use and HIV infection among MSM.

## Results

### Sample characteristics at baseline

**Figure 1:**
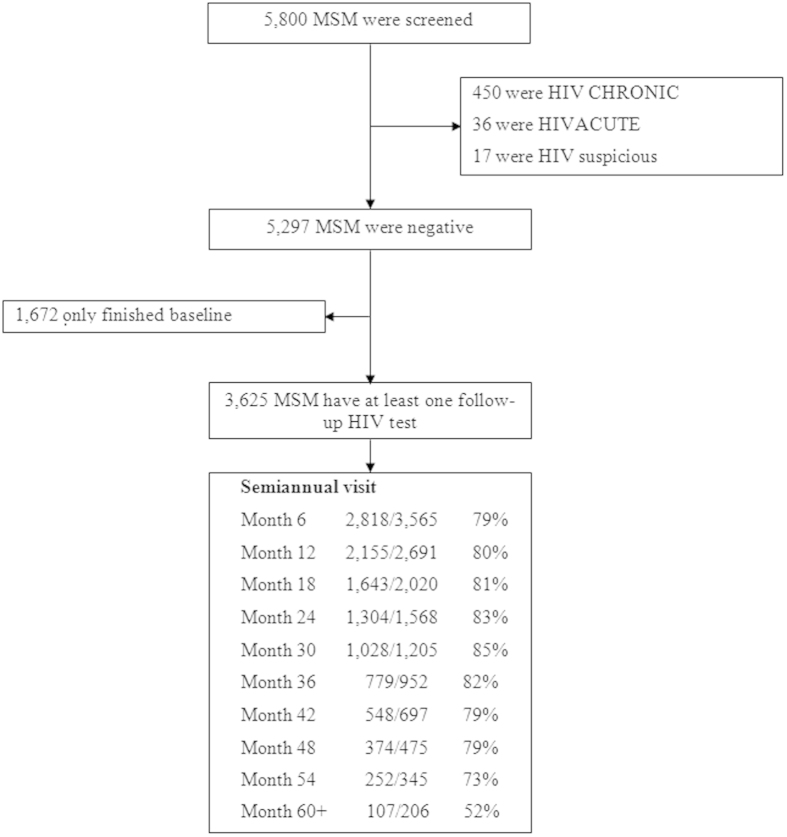
Study enrolment and follow-up.

Figure 1 shows the population sample characteristics. We screened 5,800 MSM at enrollment, of which 3,625 were HIV negative at baseline and also completed at least one follow-up interview. Leaving Beijing or refusal of follow up HIV tests were the major reasons for drop-out. The characteristics of 3,625 participants in follow-up were similar with those of those screened (Table S1).

In our sample, 66% (2,382/3,625) of participants were aged between 18 and 30 years and 2,466 (72%) of 3,422 had completed high school. Of the 3,480 participants who responded to the question on marital status, 2,405 (69%) had never married and 66 (1.9%) cohabitate with a man. Of the 1,923 participants who responded to the question on their sexual partnerships, 500 (26%) reported being in only regular partnerships, 685 (36%) reported in only casual partnerships and 738 (38%) reported both in regular and casual partnerships (Table 1). 541 (28%) respondents reported more than five casual partners in the past year.

### HIV burden in MSM

Among the 5,800 MSM screened, we identified 486 (8.4%) cases of HIV and 481 (8.3%) cases of syphilis (Fig. 1). The 3,625 participants contributed a total of 6,209 person-years of follow-up with median follow-up of 1.4 years (interquartile range: 0.5–2.7) and an average retention rate of 81% per 6-month interval. Among the 3,625 participants contributing to follow up, we identified 440 incident cases of HIV and 413 cases of incident syphilis, indicating an incidence rate of 7.1 per 100 person-years (95% CI: 6.4–7.7)for HIV and 8.6 (95% CI: 7.8–9.5) for syphilis (Table 1 & Table S2).

Individuals who contracted syphilis infection had a higher rate of incident HIV infection compared with those who remained syphilis-uninfected during our study time (HR: 1.86, 1.48–2.34). Individuals who were diagnosed with syphilis at enrollment showed a higher rate of incident HIV infection in comparison with those who had no syphilis at enrollment but contracted syphilis infection during follow-up (HR :1.47, 1.09–1.93). Furthermore, the latter showed a shorter duration from diagnosis of syphilis to seronegativity comparing with the former (0.57 vs. 1.2 years) (Table 2).

Younger MSM (aged 18–25 years) had a significantly higher risk of HIV infection than the older participants (26–40 years, adjusted HR: 0.71, 95% CI: 0.57–0.89; over 41 years: 0.53, 95% CI 0.35–0.79). MSM who reported a receptive role during intercourse were more likely to be infected compared with participants who exclusively assumed the penetrative role (receptive only: 1.99, 1.01–4.33; both: 2.27, 1.22–4.23). Although cohabiting with a regular partner suggested relative protection against incident HIV infection, the effect was not statistically significant (0.52, 0.19–1.40). Participants reporting having engaged in group sex did not experience a statistically significant higher risk of infection (1.09, 0.79–1.50). Participants reporting with an HIV-positive partner were not significantly more likely to become seropositive during the study period (negative partner: 0.89, 0.38–2.09). Sex with casual partners was also not associated with incident HIV infection (0.98, 0.61–1.58) (Table 1).

### Effectiveness of the intensive preventive intervention

**Figure 2:**
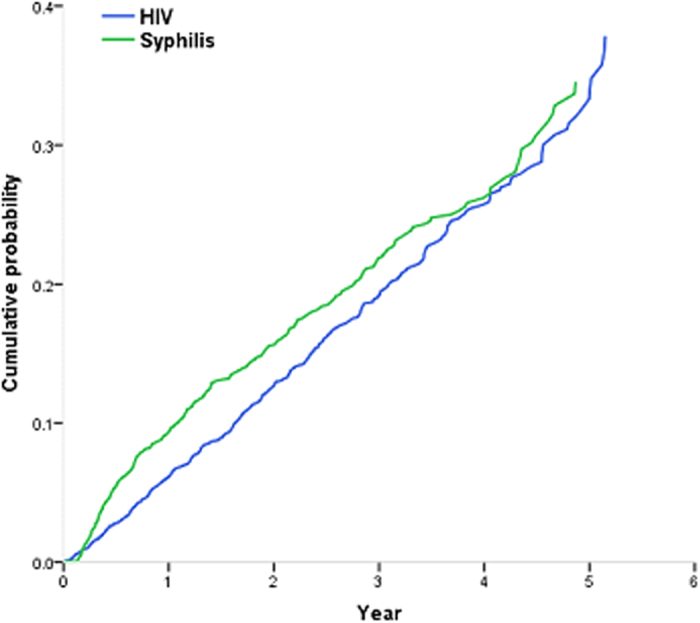
Cumulative probability of incident HIV and Syphilis infection, by number of years since enrolment.

**Figure 3:**
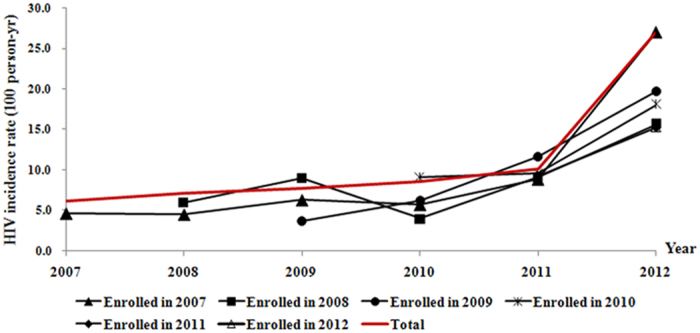
HIV infection by year for participants enrolled in the same year.

The point value for the risk rate of incident HIV infection increased during each year of follow-up, though this effect did not reach statistical significance for the first two years (RR: year 2: 1.16, 95% CI: 0.91–1.46; year 3: 1.26, 0.96–1.65; year 4: 1.40, 1.01–1.94; year 5: 1.85, 1.25–2.73) (Table 3). The cumulative probability of HIV infection increased over the 5-year of follow-up, a similar trend was observed with syphilis infection (Fig. 2). MSM who maintained their participation in the cohort did not show a low risk of infection in comparison with those who drop out the cohort earlier. There were significant peaks in the last two years respectively during follow-up (year 4: 1.33, 1.05–1.68; year 5: 1.34, 1.02–1.75) (Fig. 3). Compared with MSM who were enrolled earlier in the study, later recruits experienced higher HIV incidence rates (RR: year 2008:1.26, 0.97–1.64; year 2009:1.21 , 0.93–1.59; year 2010: 1.75, 1.30–2.36 year 2011 & post: 1.93,1.36–2.73) respectively (Table S3).

Table 4 reports a sub-analysis undertaken to investigate effectiveness of the particular condom promotion intervention in reducing HIV infection rates. Of those participants in a regular partnership, 856 reported consistent condom use at enrollment while 594 participants reported consistent use over follow-up. Participants who reported consistent condom use all the time between enrollment and follow-up did not have a significant risk of HIV infection in comparison with those who reported ever (RR: 1.18, 95%CI: 0.76–1.83) or never (1.17, 0.28–4.85) using condoms. However, MSM who reported ever or never condom use at baseline and changed to report consistent condom use in follow-up showed a slightly lower incidence rate comparing with those who still reported ever or never condom use in follow-up (Table 4).

## Discussion

In this prospective study, we found a remarkably high HIV burden among MSM in Beijing both at enrollment and during follow-up. Over 5-years of observation, the rate of incident HIV infection remained high. This finding is consistent with national surveillance data^10^, but appears to diverge from the declining estimated HIV incidence in China^9^, and is much higher than the estimate published in a recent national systematic review^13^. Participants who were enrolled in the recent years showed a higher risk of infection than those who were recruited in earlier years (Table A3), which seems to contradict the optimistic reports from the large-scale HIV treatment programs in recent years^14^,^15^. The cumulative probability of HIV infection keeps increasing. MSM presenting good compliance to follow up visits over study the period did not show a decreased risk of HIV infection compared to those who dropped out earlier. All these findings strongly suggest that intensive preventive intervention undertaken so far may not have been effective in preventing HIV infections in this subpopulation.

We assumed that MSM who were compliant to followup visits and remained in the cohort over time would have a lower risk of HIV infection because we presumed that these men would be more careful about their health and would avoid or reduce risk behavior in sex as a result of regular education and counseling on HIV prevention. However, our data shows that HIV incidence may not have significantly decreased over the period of intervention (Fig. 3). This finding in fact poses a big challenge for HIV prevention in MSM. We speculate that the result might be because our study was conducted in period of expanding access to highly active antiretroviral therapy in China, which may have created the illusion that wide spread treatment would cut HIV transmission in the MSM subpopulation, prompting young men to neglect safe sex practices. A recent report states a similar concern in South Africa^16^. More qualitative research is needed to understand how young MSM in China think about HIV, how they perceive their own risk, and what types of prevention interventions would be attractive to them. Another reason for the high incidence rate over study time could be related to the increasing proportion of men aged 26 to 40 people in our sample (Figure S2). In follow-up, the trend of increasing HIV incidence appears to be driven by young MSM, particularly those who reported having more partners.

Counseling on improving regular condom use alongside education to reinforce HIV knowledge is expected to be an effective strategy for HIV prevention, as shown in early studies in other countries^17^,^18^,^19^, but we found no difference in HIV incidence rates between those participants reporting consistent condom use and those reporting some or no condom use both at baseline and at follow-up, indicating this particular condom promotion intervention may have failed to reduce HIV infection among MSM over the period studied. This finding has several possible explanations. First, although our cohort included more than 3,600 participants, the study may have been too small to have sufficient power to identify a significant difference in incidence between participants according to their reported frequency of condom use. Notably, only 23 participants reported never using condoms at baseline and follow-up (Table 4). MSM who initially reported ever or never condom use and moved to consistent condom use have low HIV incidence rate. Second, improper use or product failure because of breakage may have mitigated effect of condom use^20^,^21^. Third, frequency of condom use relied on self-reporting, and may have been unreliable measure because of the personal nature of the questions posed and potential for desirability^22^.

Syphilis is an established risk for HIV infection^7^. In our study, we further find participants who acquired syphilis infection at follow-up were less likely to contract HIV than those who were diagnosed with syphilis at enrollment (RR: 0.68, 0.52–0.92). We hypothesize that this is due to the incident syphilis infections being diagnosed and treated more rapidly than syphilis diagnosed at baseline (mean time from diagnosis to recover: 0.5 years versus 1.23 years at baseline). It is easy to understand that syphilis screened at enrollment is usually serious than that found during follow-up. This finding indicates that test and keep syphilis free from MSM would reduce about 31 percent of HIV infection (Table 2).

Our study has several limitations. First, the participants’ frequency of condom use was ascertained by self-report, which may have resulted in overreporting of condom use frequency, bias that in turn would have weakened an association between condom use frequency and incident HIV infection. Given this bias is likely to be the same across all participants, we conclude that this particular condom promotion is ineffective at reducing HIV infection rates in this population. Second, the rate of drop out is a little bit high (about 20%) in comparison with a study on general population. However, there were no discernible differences between participants who dropped out of the study and those who remained in the study, meaning that drop out would not structurally bias the outcomes (Table S1). Third, in this study we did not investigate the impact of drug use on sexual behavior.. However, another ongoing study among MSM has shown that a large proportion of respondents reported occasional use of crystal methamphetamine and rush popper (The data of the ongoing study held with Jia), which is believed to increase sexual drive and reduce pain from anal intercourse^23^,^24^. Finally, even though we try to improve the recruitment methods, our recruitment may still have introduced some bias because of clustered features of this population.

Although several Chinese observational studies on HIV incidence in MSM exist, no cohort was observed for more than one year^25^,^26^,^27^. Those studies carried out in Beijing reported incidence rates in MSM from 3.5 to 8.1 per 100 person-years over a period of less than 1 year^11^,^12^,^13^. In the largest recent study, 48 incident cases of HIV infection were detected among 797 MSM at any follow-up over 592.98 person-years^12^. Our study’s estimate of incidence is based on the largest MSM cohort conducted in Beijing to date, with the longest follow-up. While the exact effect of condom promotion might be affected by self-reporting, the results from this cohort demonstrate that the HIV incidence rate among MSM remains high during the 5-year study, despite our intensive preventive intervention. This indicates that the current preventive strategies are not successful in preventing HIV infection in this population, and new policies of HIV prevention targeted MSM population, such as STD treatment and pre-exposure prophylaxis, should be considered.

## Methods

### Ethical Issues

The study protocol was approved by the Beijing You’an Hospital Research Ethics Committee. All study participants provided written informed consent. The methods were carried out in accordance with relevant guidelines and regulations.

### Study population and Design

A pilot study was launched in October 2006 and formal enrollment took place from May 1^st^, 2007 to December 31^st^ 2012^28^. We recruited MSM in 16 districts (18 districts before 2010) in Beijing, China (Figure S1). We mobilized, trained and hired some MSM who like to distribute flyers promoting recruitment into the study at venues frequented by MSM, including clubs, bars, parks, and bathhouses. Participants were also encouraged to refer their peers to enroll in the study. Only those respondents aged18 years or older who reported anal intercourse in the previous 6 months, and who reported having received no previous serological test for HIV antibodies or having a seronegative result were eligible for inclusion. Enrolled MSM were encouraged to return for study visits every 3 months.

A questionnaire on sexual behaviors was administered in a private room in You’an hospital by two professional medical investigators who had been trained about MSM health and sex issues before started this study. The questionnaire was administered at baseline and every 3 months.

Each participant was assigned an identifiable number. The link between a number and a specific participant was blinded for everyone except for the two investigators and PI of the study. HIV antibody and syphilis tests were identified by the unique identifiable number. HIV antibody and syphilis test were informed to participants within a week of each visit.

### Intensive preventive interventions

An intensive prevention intervention was delivered to all HIV-negative participants every three months after enrollment. The package included HIV antibody and syphilis tests (and the provision of treatment for MSM with syphilis infection), transportation allowance of 50 CNY (USD 7.40), a standardized questionnaire on sexual behaviors, ten free condoms and lubricant, education and counseling. Education and counseling sought to: enhance knowledge of HIV, teach about how to use condoms and the role of condoms to prevent HIV acquisition; clarify the different levels of risk of different sexual acts; explain the effect of STIs (such as syphilis) on HIV infection; and counsel on the importance of a regular partnership. In addition to the education and counseling provided at every follow up visit, counseling was also available by phone 24 hours a day.

### Laboratory testing

Standard HIV type 1 (HIV-1) ELISA (Abbott recombinant HIV-1/2 3rd generation, Vironostika HIV Uni-Form II plus O) and western blot analysis (Gene labs, HIV Blot2.2, AE2029) kits were used for antibody tests. Plasma HIV-1RNA copies were measured by a quantitative RT-PCR HIV-1 RNA test (Roche Cobas Amplicor HIV-1 Monitor Test). Samples that tested RNA positive with HIV-1 copy numbers below the limit of quantification (400 copies/ml) were assigned a value of 200 copies/ml for statistical purposes. RPR (Rapid plasma reagin circle test) and TPPA (trepone ma pallidum Antibody particles agglutinate experiment) were jointed used for diagnosis of syphilis.

Blood samples were evaluated at the You’an Hospital laboratory to detect seroconversion events and test results were confirmed by the Beijing Municipal Center for Disease Control and Prevention. HIV-seropositive participants were provided psychological support and referred to an acute infection cohort or care center if they met the eligibility criteria for treatment.

### Outcome Measures

The primary outcomes were HIV burden at initial screening and during follow-up. Association between compliance to follow up visits and treatment of syphilis and HIV infection were examined. HIV infection rates were also calculated by different reported frequencies of condom use. We presume that a series of intensive prevention intervention would be effective in reducing HIV infection among MSM, so we did not include a control group in the design of intervention as this would have been unethical. Self-reporting frequencies of condom were used as proxy for condom use, since actual rates of condom use could not be ascertained. Rates of infection were stratified by participants’ characteristics at enrollment, including sex role, age, education, marital status, syphilis infection status, type of partnership, prior engagement in group sex, knowledge of HIV, and HIV status of any partners. While the date of HIV seroconversion was defined as the date of clinical diagnosis for acute cases, we used the mid-point between the last negative test date and the first positive test date for chronic cases because we could not give an exact time point of seroconversion for such cases.

### Statistical Analysis

We estimated the prevalence of HIV and syphilis infections by dividing the number of HIV infections and syphilis infections at enrollment by the total number of screened participants. We calculated the incidence of HIV over the 5 years of follow-up by dividing the number of infections by the total number of person-years in that year. We used the Kaplan–Meier method to calculate the survival probabilities for the remaining HIV-negative MSM in each year. Participants who were lost to follow-up or died were regarded right-censored.

To estimate association between compliance to follow up visits and HIV incidence, we separated participants by years of follow-up to test whether the longer participants in the cohort was associated with declining rate of HIV infection over time. We estimated Cox proportional hazard ratios (HRs) for a range of factors including sex role, age, level of education, marital status, syphilis infection status, type of partnership, prior engagement in group sex, knowledge of HIV, and HIV status of partners at enrollment to determine their effect on risk of infection.

To investigate the effectiveness of condom promotion for prevention, we did a sub-analysis by using a Poisson regression model to compare rates of HIV infection among MSM reporting different frequencies of condom use (consistent: reported condom use 100%; ever: reported 1- 99%; never: reported 0%) with regular partnership (defined as a partnership that was maintained from recruitment through follow up).

A two-sided p-value of ≤0.05 was considered statistically significant. Data were double-checked using both a relational database designed in MySQL Server version 5.1and SPSS version 20.0 (SPSS, Inc., Chicago, IL, USA). All statistical analyses were performed in SPSS20.0 and SAS 9.1(SAS Institute Inc).

## Additional Information

**How to cite this article**: Jia, Z. *et al.* HIV burden in men who have sex with men: a prospective cohort study 2007-2012.. *Sci. Rep.* **5**, 11205; doi: 10.1038/srep11205 (2015).

## Supplementary Material

Supplementary Information

## Figures and Tables

**Table 1:** Incidence rate by characteristics at baseline.

**Total**	**MSMs**	**Events**	**Person-yr**	**Rate (95% CI)**	**AHR (95% CI)**
	**3625**	**440**	**6208.53**	**7.1 (6.4-7.7)**	
Age (years)					
18–25	1512	196	2280.34	8.6 (7.4–9.8)	1.00
26–40	1746	206	3188.88	6.5 (5.6–7.3)	0.71 (0.57–0.89)†
41–	367	38	739.31	5.1 (3.5–6.8)	0.53 (0.35–0.79)†
Education					
Primary school	127	13	185.77	7.0 (3.2–10.8)	1.00
Secondary school	829	105	1456.53	7.2 (5.8–8.6)	0.90 (0.51–1.61)
High school	1317	181	2304.21	7.9 (6.7–9.0)	0.97 (0.55–1.71)
Post–high school	1149	118	1946.42	6.1 (5.0–7.2)	0.74 (0.42–1.33)
Missing data	203	23	315.59	7.3 (4.3–10.3)	0.95 (0.45–2.01)
Marital status					
Never married	2405	291	3924.01	7.4 (6.6–8.3)	1.00
Married (with woman)	827	100	1612.16	6.2 (5.0–7.4)	0.99 (0.76–1.30)
Cohabit(with man)	66	4	99.10	4.0 (0.1–8.0)	0.52 (0.19–1.40)
Divorce&widower	182	27	361.68	7.5 (4.6–10.3)	1.22 (0.80–1.87)
Missing data	145	18	211.58	8.5 (4.6–12.4)	1.68 (0.91–3.10)
HIV status of partners					
Positive	41	6	60.63	9.9 (2.0–17.8)	1.00
Negative	335	48	633.79	7.6 (5.4–9.7)	0.89 (0.38–2.09)
Unknown	2337	285	3902.60	7.3 (6.5–8.2)	0.84 (0.37–1.90)
No partner	558	62	936.56	6.6 (5.0–8.3)	0.71 (0.30–1.66)
Missing data	354	39	674.95	5.8 (4.0–7.6)	0.62 (0.25–1.51)
Sex Role					
Insertive only	173	11	282.40	3.9 (1.6–6.2)	1.00
Receptive only	102	16	188.39	8.5 (4.3–12.7)	2.00 (0.92–4.36)
Both	917	124	1320.44	9.4 (7.7–11.0)	2.27 (1.22–4.23)
Missing data	2433	289	4417.30	6.5 (5.8–7.3)	1.88 (0.94–3.75)
Type of partnership					
Casual only	685	69	999.00	6.9 (5.3–8.5)	1.00
Regular only	500	52	691.58	7.5 ((5.5–9.6)	0.98 (0.61–1.58)
Both	738	99	1051.75	9.4 (7.6–11.3)	1.23 (0.79–1.91)
Missing data	1702	220	3466.20	6.3 (5.5–7.2)	1.00 (0.58–1.75)
Group sex					
No	1747	194	2487.01	7.8 (6.7–8.9)	1.00
Yes	366	50	547.87	9.1 (6.6–11.7)	1.09 (0.79–1.50)
Missing data	1512	196	3173.65	6.2 (5.3–7.0)	0.52 (0.25–1.10)
Knowledge					
Know well	326	34	503.59	6.8 (4.5–9.0)	1.00
A little	1675	197	2383.34	8.3 (7.1–9.4)	1.19 (0.82–1.72)
Unknown	175	17	269.12	6.3 (3.3–9.3)	0.93 (0.51–1.69)
Missing data	1449	192	3052.48	6.3 (5.4–7.2)	1.66 (0.69–4.00)
Syphilis					
No	3144	342	5372.28	6.4 (5.7–7.0)	1.00
Yes	481	98	836.24	11.7 (9.4–14.0)	1.86 (1.48–2.34)†

^†^indicates significant at p<0.05.

Rate: HIV incident rate.

**Table 2:** Effect of syphilis treatment on HIV infection.

**Syphilis**	**MSM**	**Duration from treatment to seronegativity of syphilis (year)**	**Rate (95% CI)**	**RR (95% CI)**
Baseline	481	1.23	11.7 (9.4–14.0)	1
Follow-up	413	0.57	8.1 (6.4–9.7)	0.68 (0.52–0.92)

Rate: HIV incident rate.

**Table 3:** HIV infection by year in period of follow-up.

**Total**	**MSM**	**Event**	**Person-yr**	**Rate (95% CI)**	**RR (95% CI)**
	**3625**	**440**	**6208.53**	**7.1(6.4–7.7)**	
Period of follow up					
≤1 year	3625	173	2810.56	6.2 (5.2–7.1)	1.00
>1 to ≤2 years	2072	115	1615.73	7.1 (5.8–8.4)	1.16 (0.91–1.46)
>2 to ≤3 years	1245	77	993.67	7.7 (6.0–9.5)	1.26 (0.96–1.65)
>3 to ≤4 years	748	46	533.23	8.6 (6.1–11.1)	1.40 (1.01–1.94)
>4 years	357	29	255.33	11.4 (7.2–15.5)	1.85 (1.25–2.73)

Rate: HIV incident rate.

**Table 4:**
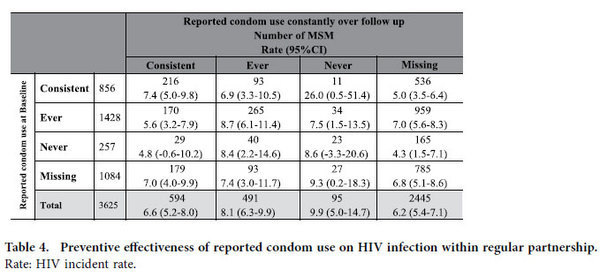
Preventive effectiveness of reported condom use on HIV infection within regular partnership.
