# Supplementary material for: HIV burden in men who have sex with men: a prospective cohort study 2007–2012
Source: Sci Rep. 2015 Jul 2;5:11205. doi: 10.1038/srep11205 (PMC5393284; doi:10.1038/srep11205)

**Supplementary Information**

**HIV burden in men who have sex with men: a prospective cohort study, 2007-2012**

Zhongwei Jia^1, 2^†, Xiaojie Huang^3^, Hao Wu^3*^, Ning Li^3*^, Tong Zhang^3*^, Peipei Ding^4^, Yixuan Sun^1^, Zhiying Liu^3^, Feili Wei^3^, Hongwei Zhang^3^, Yanmei Jiao^3^, Yunxia Ji^3^, Yonghong Zhang^3^, Caiping Guo^3^, Wei Li^3^, Danlei Mou^3^, Wei Xia^3^, Zhen Li^3^, Dexi Chen^3^, Huiping Yan^3^, Xinyue Chen^3^, Jinkou Zhao^5^, Kathrine Meyers^6^, Ted Cohen^7^, Kenneth Mayer^8^, Joshua Salomon^9^, Zuhong Lu^10*^, Christopher Dye^11^

**Table S1. The baseline characteristics of participants in follow-up and enrollee at screening**

|  | **Sample size** | | **Total** | |
| --- | --- | --- | --- | --- |
| **Age (years)** | **Number** | **%*** | **Number** | **%*** |
| 18-25 | 1512 | 42 | 2578 | 44 |
| 26-40 | 1746 | 48 | 2667 | 46 |
| 41- | 367 | 10 | 555 | 10 |
| **Education** |  |  |  |  |
| Primary school | 127 | 4 | 138 | 2 |
| Secondary school | 829 | 23 | 812 | 14 |
| High school | 1317 | 36 | 1682 | 29 |
| Post-high school | 1149 | 32 | 2893 | 50 |
| Missing data | 203 | 6 | 276 | 5 |
| **Marital status** |  |  |  |  |
| Never married | 2405 | 66 | 3912 | 67 |
| Married (with woman) | 827 | 23 | 1257 | 22 |
| Cohabit(with man) | 66 | 2 | 128 | 2 |
| Divorce & widower | 182 | 5 | 306 | 5 |
| Missing data | 145 | 4 | 197 | 3 |
| **HIV status of partners** |  |  |  |  |
| Positive | 41 | 1 | 66 | 1 |
| Negative | 335 | 9 | 509 | 9 |
| Unknown | 2337 | 64 | 3944 | 68 |
| No partner | 558 | 15 | 829 | 14 |
| Missing data | 354 | 10 | 452 | 8 |
| **Sex Role** |  |  |  |  |
| Insertive only | 173 | 5 | 243 | 4 |
| Receptive only | 102 | 3 | 153 | 3 |
| Both | 917 | 25 | 1312 | 23 |
| Missing data | 2433 | 67 | 4091 | 71 |
| **Type of partnership** |  |  |  |  |
| Casual only | 685 | 19 | 1227 | 21 |
| Regular only | 500 | 14 | 899 | 16 |
| Both | 738 | 20 | 1106 | 19 |
| Missing data | 1702 | 47 | 2568 | 44 |
| **Group sex** |  |  |  |  |
| No | 1747 | 48 | 2521 | 43 |
| Yes | 366 | 10 | 526 | 9 |
| Missing data | 1512 | 42 | 2753 | 47 |
| **Knowledge** |  |  |  |  |
| Know well | 326 | 9 | 522 | 9 |
| A little | 1675 | 46 | 2532 | 44 |
| Unknown | 175 | 5 | 234 | 4 |
| Missing data | 1449 | 40 | 2512 | 43 |
| **Syphilis** |  |  |  |  |
| No | 3144 | 87 | 5040 | 87 |
| Yes | 481 | 13 | 760 | 13 |

**Note: %* = the proportion of each level in one characteristic**

**= figure of one level / total of one characteristic**

**Table S2. Syphilis infection by year in period of follow-up**

|  | **MSMs** | **Syphilis-Event** | **Person-yr** | **Rate ( 95% CI )** |
| --- | --- | --- | --- | --- |
| **Total** | **3625** | **413** | **4776.16** | **8.6 (7.8-9.5)** |
| **Period of follow up** |  |  |  |  |
| ≤1 year | 3144 | 230 | 2345.46 | 9.8 (8.5-11.1) |
| >1 to ≤ 2 years | 1648 | 91 | 1229.95 | 7.4 (5.9- 8.9) |
| >2 to ≤ 3 years | 907 | 54 | 703.51 | 7.7 (5.6- 9.7) |
| >3 to ≤ 4 years | 508 | 21 | 347.39 | 6.0 (3.5- 8.6) |
| >4 years | 221 | 17 | 149.83 | 11.3 (6.0-16.7) |

**Table S3. HIV infection by enrolled year**

|  | **MSMs** | **HIV Event** | **Person-yr** | **Rate ( 95% CI )** | **RR ( 95% CI )** | **P-value** |
| --- | --- | --- | --- | --- | --- | --- |
| **Total** | **3625** | **440** | **6208.52** | **7.1 (6.4-7.7)** |  |  |
| **Enrolled year** |  |  |  |  |  |  |
| 2007 | 924 | 115 | 2050.81 | 5.6 (4.6-6.6) | 1.00 |  |
| 2008 | 774 | 113 | 1596.58 | 7.1 (5.8- 8.4) | 1.26(0.97-1.64) | 0.08 |
| 2009 | 801 | 98 | 1441.43 | 6.8 (5.5- 8.1) | 1.21(0.93-1.59) | 0.16 |
| 2010 | 527 | 70 | 712.22 | 9.8 (7.5-12.1) | 1.75(1.30-2.36) | 0.00 |
| 2011&2011postost | 599 | 44 | 407.48 | 10.8 (7.6-14.0) | 1.93(1.36-2.73) | 0.00 |

**Figures S1. Distribution of participants in 16 districts of Beijing**

**（Generated in ArcGIS 10）**

**
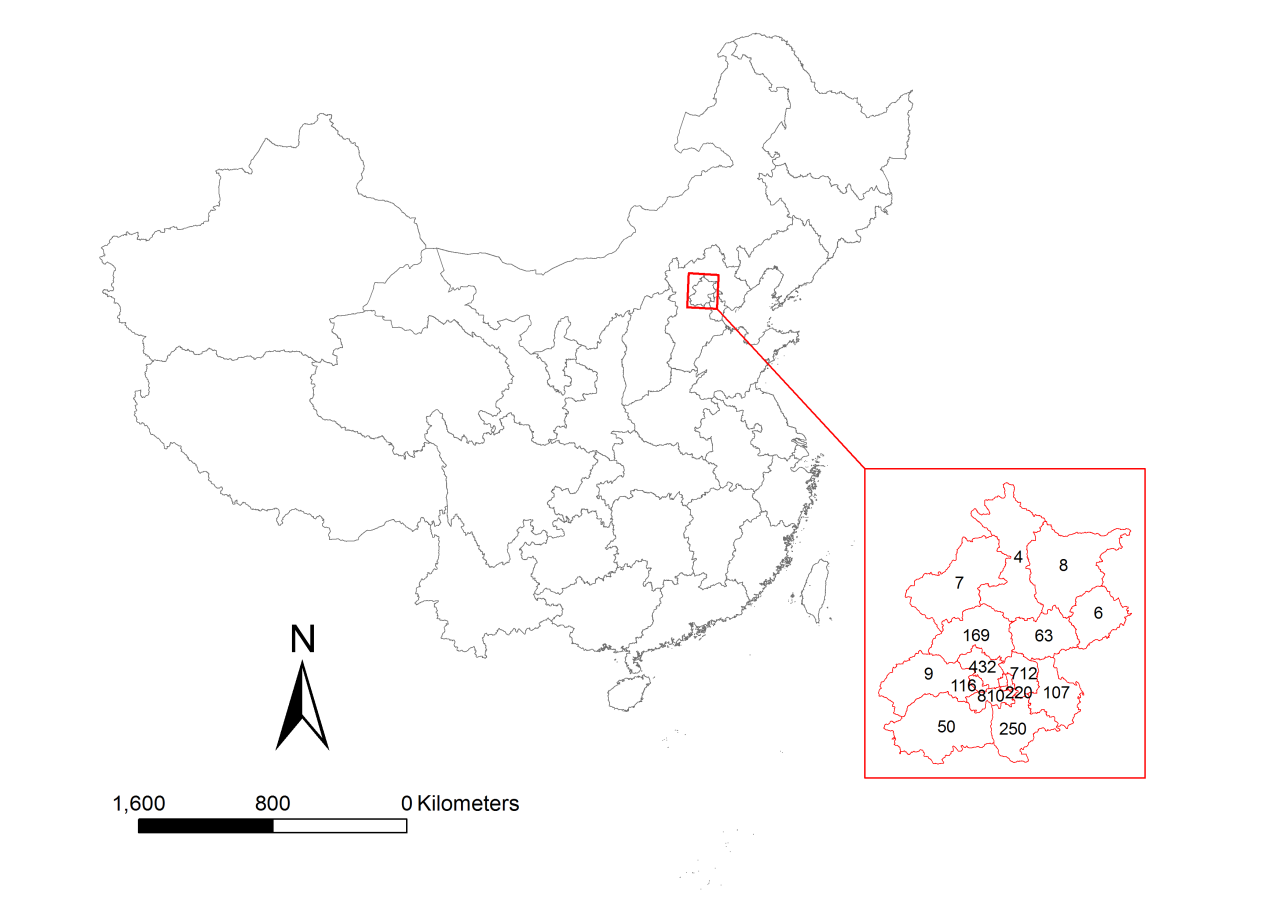
**

| **Districts** | **MSM** |
| --- | --- |
| Fengtai | 810 |
| Chaoyang | 712 |
| Haidian | 432 |
| Xicheng | 294 |
| Daxing | 250 |
| Dongcheng | 220 |
| Changping | 169 |
| Shijingshan | 116 |
| Tongzhou | 107 |
| Shunyi | 63 |
| Fangshan | 50 |
| Mentougou | 9 |
| Miyun | 8 |
| Yanqing | 7 |
| Pinggu | 6 |
| Huairou | 4 |

**Figures S2. Trend of rate of HIV infection and proportion of age group by enrolled year**


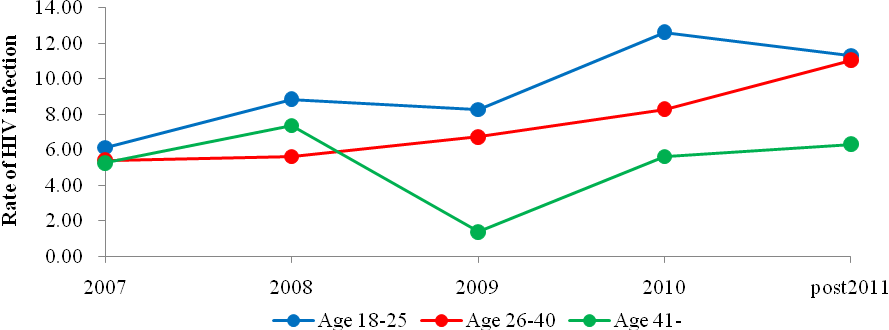


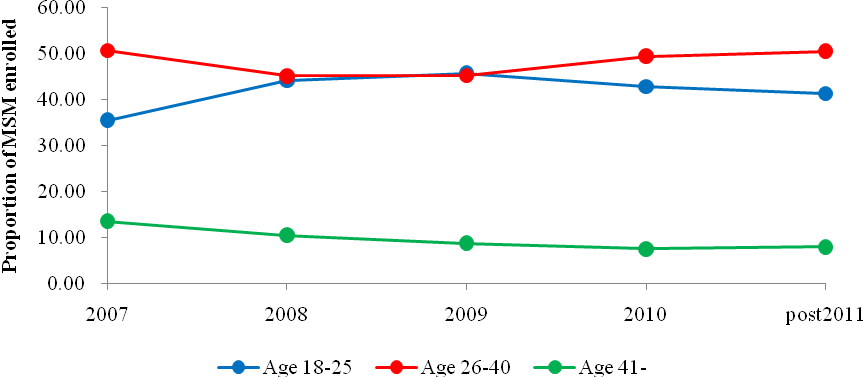

Supplement: Supplementary Information [file srep11205-s1.doc]
